# Phases of stability during major hydroclimate change ending the Last Glacial in the Levant

**DOI:** 10.1038/s41598-022-10217-9

**Published:** 2022-04-27

**Authors:** Daniela Müller, Ina Neugebauer, Yoav Ben Dor, Yehouda Enzel, Markus J. Schwab, Rik Tjallingii, Achim Brauer

**Affiliations:** 1 Section ‘Climate Dynamics and Landscape Evolution’, GFZ German Research Centre for Geosciences, Potsdam, Germany; 2 Institute of Geosciences, University of Potsdam, Karl-Liebknecht-Str. 24–25, 14476 Potsdam, Germany; 3 Geological Survey of Israel, 32 Yesha’ayahu Leibowitz, 9692100 Jerusalem, Israel; 4 The Fredy and Nadine Herrmann Institute of Earth Sciences, The Hebrew University of Jerusalem, Jerusalem, Israel

**Keywords:** Hydrology, Palaeoclimate

## Abstract

In-depth understanding of the reorganization of the hydrological cycle in response to global climate change is crucial in highly sensitive regions like the eastern Mediterranean, where water availability is a major factor for socioeconomic and political development. The sediments of Lake Lisan provide a unique record of hydroclimatic change during the last glacial to Holocene transition (ca. 24–11 ka) with its tremendous water level drop of ~ 240 m that finally led to its transition into the present hypersaline water body—the Dead Sea. Here we utilize high-resolution sedimentological analyses from the marginal terraces and deep lake to reconstruct an unprecedented seasonal record of the last millennia of Lake Lisan. Aragonite varve formation in intercalated intervals of our record demonstrates that a stepwise long-term lake level decline was interrupted by almost one millennium of rising or stable water level. Even periods of pronounced water level drops indicated by gypsum deposition were interrupted by decades of positive water budgets. Our results thus highlight that even during major climate change at the end of the last glacial, decadal to millennial periods of relatively stable or positive moisture supply occurred which could have been an important premise for human sedentism.

## Introduction

Owing to its pivotal location as the cradle of ancient cultural developments e.g.^1–3^, climatic reconstructions using Dead Sea sediments provide insights into causes for human migration, cultural rises and declines e.g.^4–6^. The last glacial-interglacial climate warming of the Northern Hemisphere was marked by multiple short and long climate fluctuations, such as Heinrich event 1 (H1), Greenland Interstadial 1 (GI-1) and Greenland Stadial 1 (GS-1)^7–9^. The most prominent expression of this climate transition in the hydroclimatically sensitive Levant is the major lake level fall by ~ 240 m^10^ of Lake Lisan and its transition into the hypersaline Dead Sea (DS) e.g.^5,11–14^, during which distinct lake level declines are recorded by massive gypsum horizons^5,11,12,15^ deposited due to water column overturn under a negative water budget^15,16^. A sequence of seasonally resolved laminations deposited between two major gypsum units—the Upper Gypsum Unit (UGU) and the Additional Gypsum Unit (AGU)—provides unprecedented insights into the in situ response of local hydroclimatic fluctuations during a major reorganization of global climate. The deposition of alternating aragonite detritus laminae between the two gypsum units^15–17^ indicates a positive water budget, and a relatively higher lake level stand^11^ lasting approximately one millennium^10^. While the mechanism for gypsum formation during falling water levels was established e.g.^13,15^, the corresponding time interval in between the two major gypsum units, and its climatic implications remain unknown.

**Figure 1:**
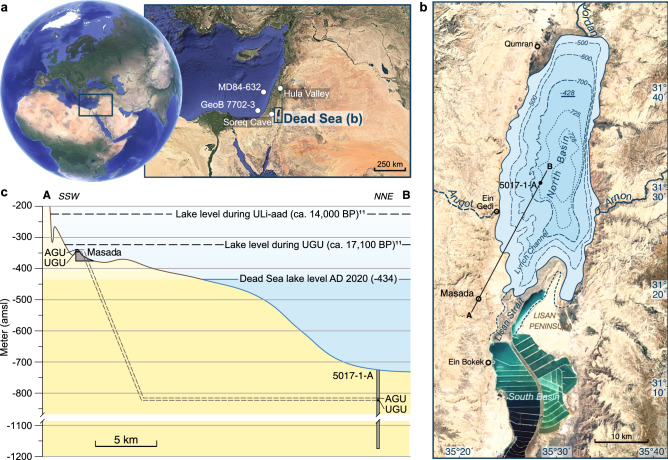
Dead Sea location, sampling sites and lake levels of the investigated time interval. (**a**) Satellite images (Map data: Google Earth, SIO, NOAA, U.S. Navy, NGA, GEBCO) of the location of the Dead Sea and other sites (Soreq Cave^18^, Hula Valley^19^, MD84-632^20^, GeoB 7702-3^21^) discussed in this study. (**b**) Bathymetric map and satellite image (Map data: Google Earth, SIO, NOAA, U.S. Navy, NGA, GEBCO) of the Dead Sea with sampling sites onshore (Masada) and in the deep lake center (core 5017-1-A). (**c**) Topographic/ bathymetric profile from Masada to 5017-1-A. Approximate lake levels during the study interval are from Torfstein et al.^11^, and are shown in comparison to the water level in AD 2020 (in meter above mean sea level, amsl). The dashed lines connect the Upper Gypsum Unit (UGU) and Additional Gypsum Unit (AGU; see “Methods”) at both sites.

**Figure 2:**
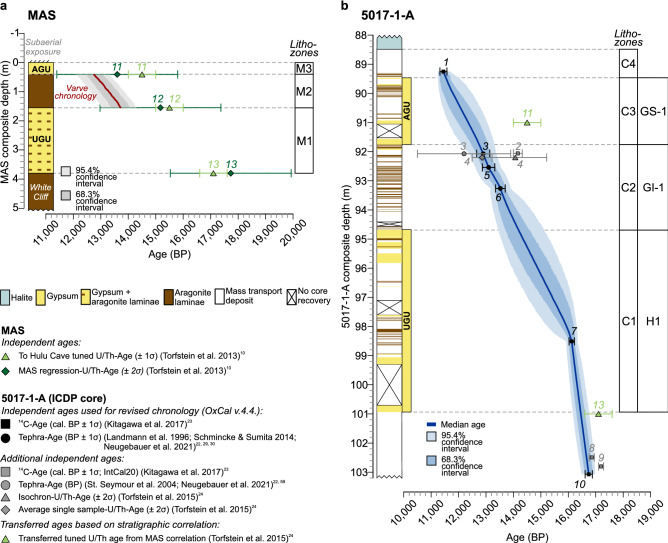
Age model and published ages for Masada (MAS) and the ICDP core (5017–1-A). (**a**) Published U/Th ages for Masada^10^ and varve chronology (this study). The MAS varve chronology is anchored at the base of the AGU and provides a minimum estimate for the time interval between the UGU and AGU. Simplified lithology on the left. (**b**) Bayesian age depth model constructed with OxCal^26–28^ (“Methods”) for the ICDP core. Numbering of ages according to Table S1. Black colored ages are included in the age model. Simplified lithology on the left. See “Methods” for discussion of ages excluded from the age modelling. *AGU* Additional Gypsum Unit, *UGU* Upper Gypsum Unit, *H1* Heinrich event 1, *GI-1* Greenland Interstadial 1, *GS-1* Greenland Stadial 1.

Here, we compare marginal lake sediments from the Masada outcrop located at the southwestern shore of the DS with deep-lake facies from core 5017-1-A of the ICDP Dead Sea Deep Drilling Project (DSDDP)^12^, deposited between the UGU and AGU in order to analyze millennial-scale climate changes at high-resolution (Figs. 1, 2, “Methods”). Both segments cover the end of the Lisan Formation, before its deposition at Masada terminated due to the lake level decline, whereas the transition into the Holocene is recorded only by ICDP core 5017-1-A retrieved from the depocenter of the lake.

In this study, we provide new microfacies and sub-millimeter XRF analyses from marginal (Masada) and deep-water (core 5017-1-A) sediments (“Methods”) and use these to reconstruct hydroclimatic variability partly at annual resolution during the final stage of Lake Lisan between ca. 17 and 11 ka before its transition into the DS. We further provide a revised Bayesian age model (“Methods”) for the Lateglacial using recently published tephra ages^22^ and radiocarbon dates^23^ in comparison to previous U/Th dating^10,24^.

## Results

### Chronology and age model

The published Masada chronology for the AGU and UGU is based on U/Th- and radiocarbon dates, which were tuned to the U/Th chronology of the Hulu Cave speleothem record in China^10^ (Fig. 2a, Table S1). Based on lithological correlation, these tuned ages for the base of the AGU (14.5 ± 0.5 ka), and the UGU (15.5–17.1 ± 0.5 ka) were transferred from the marginal outcrops to the ICDP core^24,25^ (Fig. 2b). The recent finding of lateglacial cryptotephra mainly from well-dated eastern Anatolian volcanic eruptions^22^ directly in the ICDP core, however, requires a partial revision of previous chronologies. We consider the tephrochronological dating as robust and apply three tephra-derived ages together with three published radiocarbon dates (one above the AGU, one in the middle of the UGU and one from the base of the UGU; Fig. 2b)^23^ for Bayesian age depth modelling in OxCal^26–28^ for the lateglacial ICDP core (Fig. 2b, “Methods”). The revised age for the base of the UGU (16,449 + 143/− 149 BP) is within uncertainties in agreement with published U/Th-ages^10^ (Fig. 2b). However, the revised chronology dates the top of the UGU at 14,186 + 394/− 459 BP and the base of the AGU at 12,753 BP + 308/− 276 BP, thus younger than in previous age models. The duration between the two gypsum units is confirmed by independent counting of almost 1000 varves in both, the ICDP core and at Masada (“Methods”). An older age for the gypsum unit as suggested by the tuned U/Th age from the Masada chronology^10^ is not plausible, because the younger tephra horizons are deposited ~ 8–10 decades before the onset of the AGU (tephra ages No. 3 and 5 in Fig. 2b). The volcanic eruption producing these tephra are reliably dated in the proximal site of Lake Van^29,30^ and further agree within dating uncertainties even with non-tuned U/Th ages obtained at similar depth in the ICDP core^24^ (No. 4 in Fig. 2b) and the regression-U/Th-age for the base of the AGU at Masada (13.6 ± 2.2 ka BP; dark green No. 11 in Fig. 2a)^10^. The only disagreement remains with the tuned U/Th age for the base of the AGU from the Masada chronology (light green No. 11 in Fig. 2), which is based on the Hulu Cave record. Ages from our revised age model in the ICDP core were transferred onto the Masada chronology through lithological correlation of the gypsum units (Fig. 2a).

### Lithology of the ICDP core 5017-1-A

**Figure 3:**
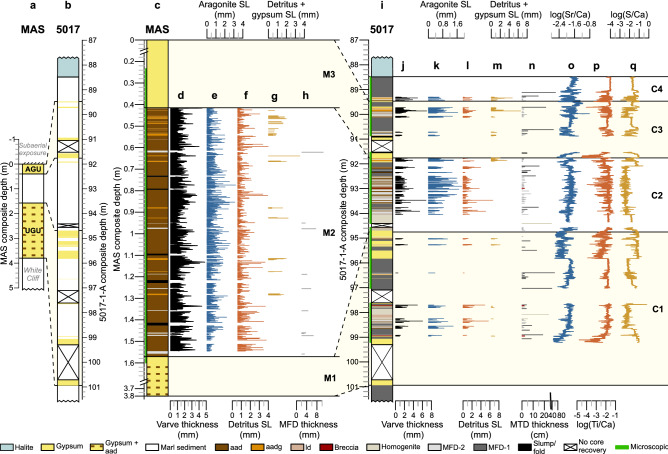
Sediment profiles at Masada and in the ICDP core. Overview of study intervals at Masada (**a**, MAS) and the deep ICDP core (**b**, 5017) on the same scale intervals for thickness comparison (see Fig. 2 for chronological details). UGU: Upper Gypsum Unit, AGU: Additional Gypsum Unit. (**c**–**h**) Zoom in at the MAS study interval: (**c**) lithological column, (**d**) varve thickness (mean 1.1 mm), (**e**) aragonite, (**f**) detritus and (**g**) detritus + gypsum sublayer (SL) thicknesses, (**h**) thickness of MFDs and lithozones M1–M3. (**i**–**q**) Study interval of the ICDP core: (**i**) lithology, (**j**) varve thickness (mean 0.82 mm), (**k**) aragonite, (**l**) detritus and (**m**) detritus + gypsum sublayer (SL) thicknesses, (**n**) thickness of MTDs, (**o**) log(Sr/Ca), (**p**) log(Ti/Ca) and (**q**) log(S/Ca) ratios and lithozones C1–C4.

Four lithozones (C1-C4) with sharp transitions were distinguished (Fig. 3i–q, Table S2) in the ~ 12 m long section of the ICDP core. The following microfacies descriptions are confirmed by (µ-) XRF measurements (Fig. 3o–q, Supplementary Discussion 4). The sections of the ICDP-DSDDP core comprise (i) alternating aragonite and detritus (aad) varves, (ii) laminated and massive gypsum deposits, and (iii) mass transport deposits (MTDs)^12^ that are mostly mass flow deposits (MFD) (Fig. S1, Table S2, Supplementary Discussion 1). Aad varves are formed by aragonite deposition during summer and detrital supply by winter floods e.g.^12,17,31^ and are characterized by high log(Sr/Ca) and log(Ti/Ca) ratios reflecting the two alternating sublayers^31–33^. On the other hand, gypsum deposits are indicated by high log(S/Ca) ratios^31–33^. Ages in the following refer to the revised age model of the ICDP core using radiocarbon^23^ and tephra^22^ ages (Fig. 2, Table S1, “Methods”).

Lithozone C1 is coeval with the UGU and comprises seven discrete gypsum intervals (Fig. S1a-h, u) intercalated with mm- to dm-scale, often erosive, MTDs (Fig. S1q-t) and a total of ~ 340 aragonite varves (Fig. S1i-p). The gypsum beds are predominantly laminated (facies gd-l1: Fig. S1a-h, u, Supplementary Discussion 1.1, 3), and primarily composed of alternating gypsum and detrital sublayers (Fig. S1a-d) suggesting gypsum formation in the water column during summer due to evaporation causing water level lowering^15,16,34–36^ and surface runoff in the rainy winter season providing detrital influx. Gypsum precipitation is indicated by interlocking rectangular grain shapes, which have been interpreted as characteristic for gypsum precipitation from lake water^34–36^. Occasional inverse grading of gypsum sublayers indicates slowing crystal growth with ongoing evaporation e.g.^34^ thus supporting this interpretation. However, since no modern analogue for the observed laminated gypsum facies exists at the Dead Sea, its formation processes are difficult to prove and reworking cannot be fully excluded. Gypsum intervals are interrupted by four ~ 30–230 varve-long phases of aragonite varves and MTDs including sequences of up to ~ 40 varves. These varves are dominated by alternating aragonite and fine-grained detritus (‘aad’ varves^12,17^; Fig. S1i-l) with subordinated ‘aadg’ (Fig. S1m-p) and ld-type (laminated detritus^12,37^) varves (Supplementary Discussion 1.1, 1.2). Gypsum in ‘aadg’ varves is reflected in elevated log(S/Ca) ratios with similar values as measured in the gypsum beds and occurs either within the detrital or as separate sublayer following aragonite sublayers. Therefore, we cannot unambiguously distinguish reworking^34,38^ or precipitation^34–36^ as causes for gypsum in ‘aadg’ varves. Since diagentic crystal growth structures are rare, post-depositional formation is, if at all, subordinated.

**Figure 4:**
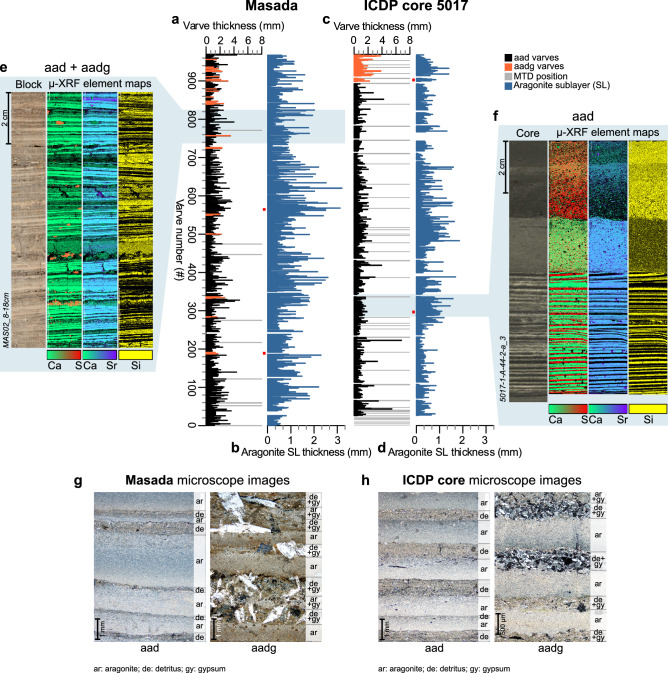
Aragonite varves of the ULi-aad. Varve (**a**) and aragonite sublayer (SL) thicknesses (**b**) for Masada and varve (**c**) and aragonite sublayer (SL) thicknesses (**d**) for the ICDP core. In the varve thickness plots (**a**,**c**) aadg varves are marked in orange and MTDs as grey bars. Gaps in the ICDP core (**c**,**d**) are due to erosion of varves by MTDs and were estimated from parallel Masada varve counts. Gaps in the Masada profile (**a**,**b**) are due to folded varves that were counted without thickness measurements. Note: two phases of increased aragonite sublayer thickness at both sites (**b**,**d**). Red points in (**a**,**c**) mark position of thin section images (**g**,**h**). Blue shading indicates position of 2D µ-XRF element maps (**e**,**f**) of resin-impregnated sediment blocks: (**e**) Prolonged period of 89 continuous varve formation at Masada and (**f**) 54 years of calm and undisturbed aragonite varve formation followed by MTDs in the ICDP core. Aragonite sublayers (blue colors in Ca + Sr maps) alternate with detrital sublayers (yellow Si maps) in aad varves. Orange colors in Ca + S maps indicate gypsum. (**g**,**h**) Microscope images of aad and aadg varves from Masada (**g**) and the ICDP core (**h**). Note the different scales.

Lithozone C2 comprises ~ 910 aad/aadg varves (Fig. 4c,d,f,h, S1i-p, Supplementary Discussion 1.2, 2) intercalated with mm- to cm-scale, mostly non-erosive MFDs (Fig. S1t; C2 hereafter labelled as ULi-aad–Upper Lisan aad varves). The basal ~ 50 aad varves are frequently intercalated with MFDs, which distinctly decrease in number upcore, where sequences of up to ~ 60 varves without any intercalated MFD are shown by both microscopic inspection (Fig. 4c) and novel high-resolution µ-XRF mapping (Fig. 4f). The upper varved part is interrupted by a series of primary^34–36^ gypsum laminae (facies gd-l1: Fig. S1a-h, u, Supplementary Discussion 1.1, 3) followed by ~ 60 aadg varves marking the top of this lithozone (Figs. 4c, S1m-p). Two ca. 110 and 200-year long phases of significantly thicker aragonite sublayers occur during this varved interval (Table S3).

Lithozone C3 corresponds to the AGU and includes four gypsum beds (Fig. S1a-h, u) intercalated with cm- to dm-scale, partly erosive MTDs (Fig. S1q-t) and a total of ~ 210 varves (Fig. S1i-p). In contrast to lithozone C1, only about half of this gypsum unit is partially laminated (facies gd-l1: Supplementary Discussion 1.1, 3)^34–36^, while the other half appears massive and potential formation processes remain elusive. Varved intervals comprise up to 40 year-long sequences of aad (Fig. S1i-l) and aadg varves (Fig. S1m-p).

The lower part of lithozone C4 comprises ~ 50 aadg varves (Fig. S1m-p) intercalated with some MFDs (Fig. S1q-t), while its upper part represents a series of dm-scale erosive MFDs. The lithozone ends with the sharp onset of halite deposition.

### Lithology at Masada

The uppermost ~ 3.8 m of the Lisan Formation at Masada represent the final deposition of Lake Lisan before sedimentation terminated at this site due to the lake level fall (~ 17–11.5 ka). Three lithozones M1–M3 (Fig. 3c–h, Table S2) comprising aragonite varves and laminated gypsum are distinguished (Fig. S2, Supplementary Discussion 1). Due to difficulties in preparation of thin sections with undisturbed structures, detailed microfacies analyses could not be carried out for lithozones M1 and M3.

Lithozone M1 corresponds to the UGU and includes nine coarsely laminated gypsum beds intercalated with sequences of aragonite varves. The upper gypsum bed comprises laminated gypsum facies gd-l2 (Fig. S2c, Supplementary Discussion 1.1).

Lithozone M2 (ULi-aad) comprises ~ 930 aad and ~ 40 aadg varves (Fig. 4a,b,e,g, aad: Fig. S2d-f, aadg: Fig. S2g, h, Supplementary Discussion 1.2, 2). Continuous sequences of up to ~ 300 varves occur with a few cm-scale, non-erosive MFDs (Fig. S2l). In the uppermost ~ 130 varves, the share of aadg varves in between regular aad varves increases (Fig. 4a), which are clearly recorded by µ-XRF mapping (Fig. 4e). In contrast to the correlating lithozone C2 in the deep-water site, gypsum in ‘aadg’ varves shows diagenetic crystal growth structures (Fig. 4g, S2g, h, Supplementary Discussion 1.1) suggesting post-depositional formation at this shallow water site. Two ca. 140-year long phases of significantly increased aragonite sublayer thickness are also seen in the Masada record (Table S3), similar as found in the ICDP core (lithozone C2), further confirming the correlation of both varved intervals C2 and M2.

Lithozone M3 is a ~ 40 cm thick pure gypsum deposit defined as AGU and consists of undulating gypsum layers. Microfacies analyses of the lowest part show laminated gypsum facies gd-l3 with undulating layer boundaries suggesting wave activity at shallow water conditions (Fig. S2a, b, Supplementary Discussion 1.1). The top of this lithozone marks the end of the sedimentary sequence at Masada and sediments corresponding to lithozone C4 in the ICDP core were either not deposited or eroded.

### Shallow versus deep-water sedimentation

The correlation of Masada and ICDP core sediments is based on the UGU and AGU observed in both records (Fig. 3a,b) and reveals several differences in our study interval. The transition into the Holocene is not present at Masada due to the falling lake level and possible erosion. This time interval is only recorded in the depocenter in lithozone C4 of the ICDP core (Figs. 2, 3). The dominance of MFDs in lithozone C4 intercalated with only ~ 50 aadg varves confirms slope instability, probably due to rapid lake level decrease or earthquakes e.g.^12,17,39,40^. Erosion of varves by the MFDs is likely, which is why the counted ~ 50 varves provide only a minimum estimate for the duration of this interval.

Another distinct difference between the two depositional environments is the increased frequency and thickness of MTDs in the depocenter. MTDs in the deep-water gypsum deposits increase the thicknesses of sections C1 and C3 by a factor of ~ 2.5 and ~ 5.5 with respect to M1 and M3 at Masada. Even the gypsum beds themselves are up to ~ 1.5 times thicker in the deep water environment likely due to longer submergence under a thicker water column^15^. At Masada, gypsum is primarily reworked or diagenetic with fine-grained detrital aggregates of pellets indicating a shoreline environment, whereas precipitated gypsum and fine-grained detritus or cement dominate the depocenter (Supplementary Discussion 1.1).

The varved ULi-aad (Fig. 4) unit bound by the two gypsum units also differs between the two sites. The frequency and thickness of MTDs is higher in the deep basin leading to a ~ 2.5 times higher sedimentation rate than at Masada (Fig. 3a,b,i). Yet, the duration of independently established floating varve chronologies is strikingly similar at both sites (M2: 968 + 15/− 64 varves; C2: 912 + 15/− 24 varves; Fig. 4a–d, Supplementary Discussion 2) indicating only minor erosion by MTDs in the deep-water site. Interestingly, mean varve thickness is 1.3 times higher in M2 compared with C2 (1.1 mm *versus* 0.82 mm, respectively) due to thicker aragonite sublayers (Fig. 4b,d; Supplementary Discussion 2). Aragonite sublayers in the shallow-water site are likely thicker because of the site’s proximity to freshwater inflow, inhomogeneous aragonite accumulation in the basin^41^ and/or dissolution at the depocenter. In both sites, the two oscillations of higher aragonite sublayer thickness lasting between ~ 110 and ~ 200 years (Fig. 4b,d; Supplementary Discussion 2) suggest centennial-scale intervals of increased freshwater inflow that could have supported aragonite precipitation^16,42^, although other mechanisms, like calcite-rich dust or sulfate-reducing bacteria, could have contributed bicarbonate as well^41^.

## Discussion: a millennium of stability

Two discrete gypsum units formed across the basin^15^ during the major lake level decline of Lake Lisan at the end of the last deglaciation before its transition into the hypersaline DS, which were interpreted as intervals of accelerated lake level fall^11,15^ (> 100 m)^5^. However, the interval between the two gypsum units so far was not investigated in detail, although it can provide insights into the regional responses and impacts of global climate change in the eastern Mediterranean. Therefore, we here discuss the results of our high-resolution analyses of the aad facies (ULi-aad) depicting the internal structure and variability in the millennium between the two final Lake Lisan gypsum units. We further discuss internal variability within the gypsum units themselves as well as the transition into the Holocene based on our new high-resolution data from the ICDP core.

**Figure 5:**
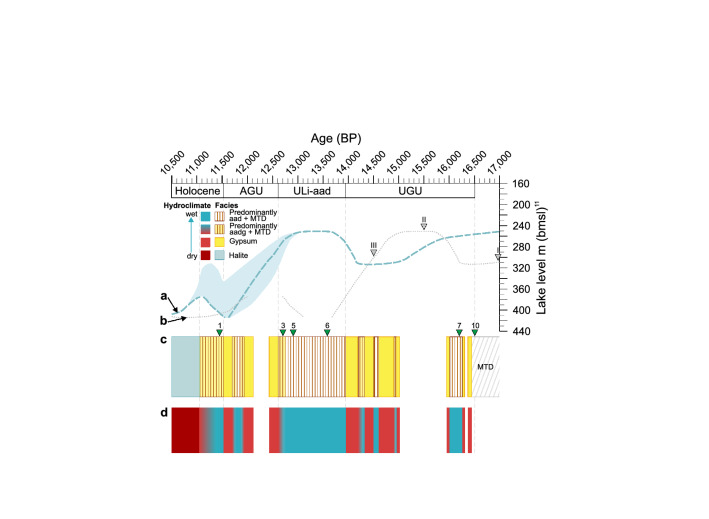
Thin section and XRF-mapping based microfacies changes and their hydroclimatic interpretation *versus* published lake-level curve. (**b**) Lake level curve (in m below mean sea level, m bmsl) from Torfstein et al. (2013)^11^ (**a**) on the updated chronology. Note that the exact timing of the lake level drop during the AGU is uncertain as displayed by the light-blue shaded background. (**b**) Tuned U/Th ages from Masada^10^ are shown by grey triangles. (**c**) Microfacies variations shown on the updated chronology. Position of age tie points from the updated chronology (Fig. 2, “Methods”) are shown by green triangles. Numbering of ages according to Fig. 2 and Table S1. Ages 1, 7 and 10 denote radiocarbon ages from Kitagawa et al.^23^, and ages 3, 5 and 6 indicate the position of tephra horizons from the Nemrut V-16 and Süphan swarm eruptions V8-V15 from Neugebauer et al.^22^. Tephra ages are from Landmann et al.^30^ and Schmincke & Sumita^29^. Yellow rectangles: gypsum beds; brown-white streaked rectangles: predominantly aad varves including intercalated MTDs; brown-yellow streaked rectangles: predominantly aadg varves including intercalated MTDs; gaps in the facies mark core gaps. (**d**) Hydroclimatic interpretation of the microfacies in (**c**). Gypsum indicates pronounced drops in lake level during negative water budgets (dry phases: red boxes) and aragonite varves indicate rising lake levels during a positive water budget (wet phases: blue boxes). Dark red rectangle: driest phase with lake level drop below ~ 400 m bmsl leading to halite deposition. *AGU* additional gypsum unit, *ULi-aad* Upper Lisan aad, *UGU* Upper Gypsum Unit.

The first ~ 50 varves following the sharp termination of the UGU are frequently intercalated with event layers (Fig. 4c) suggesting that the initial lake level rise of ~ 60 m^11^ occurred rapidly within five decades coinciding with an increased flood frequency. After this rapid lake level rise we observe intervals of up to six decades without any event layers even in the ICDP core (Fig. 4a,c), suggesting stable meromictic conditions without shore or margin disturbance nor local extreme precipitation events. Except for two intervals of increased aragonite sublayer thickness lasting ~ 110–200 years, sedimentation was exceptionally stable for about 800 years (Fig. 4a–d). Early signs of the end of this stability only occurred in the last ~ 130 years of this positive water budget phase (Fig. 5a,d), when an increasing number of aadg varves (Fig. 4a,c) and few primary gypsum laminae indicate increasing summer evaporation. According to the revised age model (Fig. 2, “Methods”), the ULi-aad coincides with the lateglacial interstadial in Greenland (GI-1)^43^ and the Bølling-Allerød^44,45^.

The deposition of the UGU gypsum has been related to the cooling in the North Atlantic during H1 (17.5–14.6 ka BP in the North Atlantic^46^)^11,47^. It has been assumed that cyclogenesis in the Mediterranean was reduced during this period and, therefore, caused dry conditions in the Dead Sea watershed^11,47^ and a lowering of the water level (Fig. 5a,b), which then led to water column overturn and gypsum formation^15,16^. However, we find that the UGU gypsum was repeatedly interrupted by four decadal- to centennial-long periods of water level rises indicated by the varved intervals (Fig. 5c,d). These findings let us assume that the lake level was not constantly at ~ 330 m bmsl or below as previously assumed^10,13,47^, but rose several times above the threshold for gypsum formation allowing for meromictic conditions required for aragonite precipitation^41^. Interestingly, varve sequences lasting up to 40-years without any intercalated event layers provide evidence for several decades of depositional stability. The absence of major events for several decades even during phases of instable lake levels is surprising and suggests knowledge gaps in our understanding of the link between climate boundary conditions and extreme event occurrence.

Following the millennium-long relatively stable high-stand, the AGU gypsum indicates a generally dry interval (Fig. 5a), interrupted once by a meromictic phase lasting ~ 210 years, during which water levels rose above the threshold for gypsum formation once more (Fig. 5c,d). Even during this period, a single ~ 40 year-long calm and stable phase without extreme events is recorded. Based on tephrochronological dating (Fig. 2), the AGU coincides with the Younger Dryas/GS-1^22^, thus indicating a generally dry spell in the Levant during this cold phase, in contrast with previous interpretations of a wet Younger Dryas^10,13^. This indicates that the climatic influence on prehistoric human development in the region, such as the rise and demise of the Natufian culture e.g.^6,19,48^, should be revisited.

Between the AGU low-stand (Fig. 5a) and the onset of halite deposition during the early Holocene (Fig. 5c), which reflects the final lake level fall below the threshold for halite deposition of ~ 400 m bmsl^13^, a short lake level rise lasting ~ 50 years (Fig. 5a) is indicated by the deposition of varves and MTDs in the deep basin (Fig. 5c,d). According to radiocarbon ages (“Methods”) this short-term rise occurred during the last glacial to Holocene transition^23^, and sediments associated with this short interval are missing in the littoral zone at Masada due to subsequent erosion or lack of deposition.

**Figure 6:**
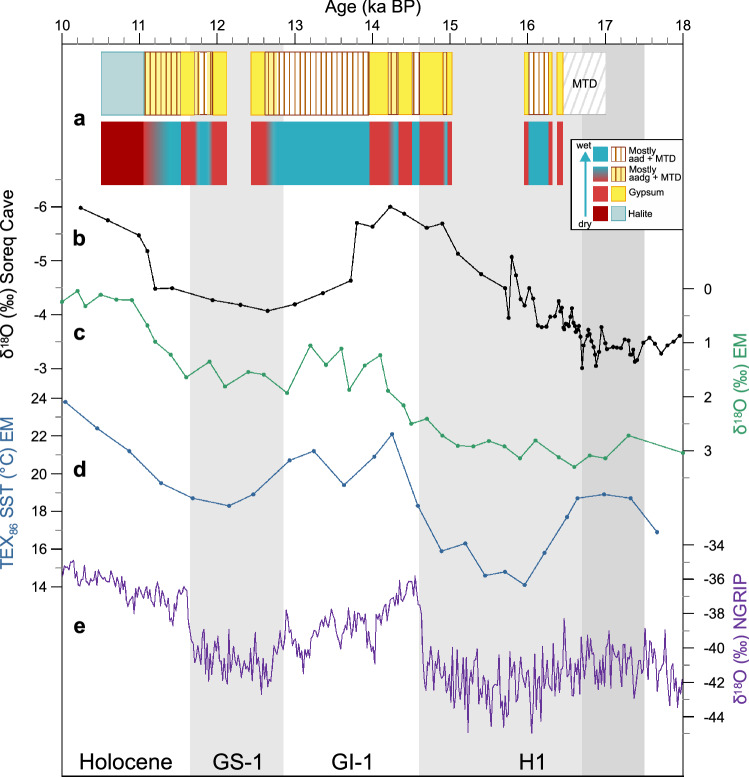
Regional comparison on original time scales. (**a**) Lithological data and related changes in water budget from this study (see Fig. 5). (**b**) δ^18^O from speleothems in Soreq Cave, Israel, from Bar-Matthews et al.^18^. (**c**) δ^18^O from *G. ruber* in core MD84-632, Eastern Mediterranean (EM) Sea, from Essallami et al.^20^. (**d**) TEX_86_-based sea surface temperature (SST) from core GeoB 7702‐3, Eastern Mediterranean Sea, from Castañeda et al.^21^. (**e**) δ^18^O from the NGRIP ice core, Greenland, on the GICC05 timescale from Rasmussen et al.^43^. Boundaries of the Greenland Stadial 1 (GS-1) in Greenland ice cores after Rasmussen et al.^43^, boundaries of Heinrich event 1 (H1) in the Ice Rafted Debris belt, North Atlantic, after Stanford et al.^46^. Note that offsets in the records might be due to the dating uncertainties in the range of several hundreds of years in all presented records. *GI-1* Greenland Interstadial 1.

In a regional context, the negative water budgets that we find in the DS during H1 and GS-1, as well as the positive water budget during GI-1, also appear in lower resolution δ^18^O data from the Soreq speleothem in the Judaean Mountains west of the Dead Sea^18^ and from planktonic foraminifera in the Levantine Sea^20^, although less distinct for H1 (Fig. 6a–c). These δ^18^O records indicate drier conditions (more positive values) during H1 and GS-1, and wetter conditions during GI-1 (more negative values). Despite the general agreement of dry conditions in the Eastern Mediterranean (EM; Fig. 6a–c) during cold periods in the North Atlantic realm (Fig. 6e), we see some differences in the timing and duration of these dry phases. In particular, the dry phase during GS-1 appears to be shorter in our record than in the marine and speleothem records (Fig. 6a–c). Since the duration of the gypsum units in the DS is difficult to determine, it remains elusive if the observed differences are due to dating uncertainties or if they reflect different local responses to climate. The timing of the onset of the UGU as a response to dry climate in the DS basin is in a good agreement with the TEX_86_-based sea surface temperature (SST) decline in the EM^21^ (Fig. 6a,d) supporting earlier reports that low SST in the EM could lead to dry conditions in the Levant^18^. The deposition of Ice Rafted Debris in the North Atlantic marking H1 started ~ 800 years earlier than cooling and drying in the EM and Levant at ~ 17.5 ka BP^46^, but due to dating uncertainties it cannot be proven if the drier conditions in the EM might be a delayed response to iceberg drift in the North Atlantic. Wetter conditions during the lateglacial interstadial as evidenced by the ULi-aad in the DS are also reported from pollen-based winter precipitation reconstructions in the Hula Valley ~ 200 km north of the DS^19^ and from other sediment records in the region including Lake Yammoûneh^49^, and Bekaa Valley^50^ likely related to warmer SSTs in the EM^21^ (Fig. 6d). The warmer EM SST mirrors the hemispheric-scale lateglacial warming, which is particularly pronounced in the NGRIP ice core record in Greenland^43^ (Fig. 6e). The subsequent cooling in Greenland (GS-1)^43^ and of the SST in the EM^21^ (Fig. 6d,e) is contemporaneous within dating uncertainties with the AGU and dry conditions at the DS (Fig. 6a). Dry conditions during the YD were also reported from Bekaa Valley^50^, the Hula Valley^19^ and a composite study of several EM sites^51^.

Our new high-resolution sediment data from the deep basin and the littoral zone of Lake Lisan reveal new insights particularly into short-term hydroclimatic variability during the Lateglacial in the Levant. It becomes evident that even during times of the large-scale climate change from glacial to interglacial conditions with major hydroclimatic consequences, decadal to centennial periods of relative stability and positive water balance occurred. This emphasizes the importance of high-resolution palaeoclimate records with seasonal information for understanding the full temporal range of climate variability in the climatically sensitive Levant. The information of decades of relative climatic stability during the Lateglacial should be also considered in assessments of the role of climate for the development of human sedentism in this region during that time.

## Methods

### Sites and sampling

The cores from site 5017–1-A were obtained from the depocenter of the Dead Sea at ~ 300 m water depth (Fig. 1; N31° 30.483ʹ E35° 28.273ʹ) during the International Continental Scientific Drilling Program Dead Sea Deep Drilling Project (ICDP-DSDDP) in 2010/2011 (details in Neugebauer et al.^12^). Drilling was performed with the Deep Lake Drilling System (DLDS) operated by the non-profit corporation DOSECC (U.S. Drilling, Observation and Sampling of the Earth’s Continental Crust)^12^. The cores comprise authigenic halite, gypsum, aragonite and clastics^12^. Especially the sedimentary sequence of the Lisan Formation (MIS2-4) is dominated by varves of alternating authigenic aragonite and allochthonous detritus (aad) e.g.^17,52^. This study focusses on the upper Lisan Formation between ~ 101 and ~ 88.5 m sediment depth from sections 5017-1-A-47-1, -46, -45, -44, -43 and -42-3 of drilling site A at the depocenter of the lake. Cores 46 to 43 (~ 9.3 m) were continuously investigated by thin section microscopy (marked by green line in Fig. 3c,i), and we provide additional macroscopic information from core catchers (cc), as well as from overlying and underlying sedimentary sections of 87.73–88.48 m (core 42–3) and 100.66–103.15 m (core 47–1) sediment depth, respectively. Five gaps occur due to no core recovery (marked with an X in the lithological profile in Fig. 3i). In lithozone C3, one gypsum bed was obtained in core catcher 43-cc.

The study interval at Masada (N31° 18.602ʹ E35° 22.489ʹ, bottom of the UGU -347 m a.m.s.l.) encompasses the uppermost Lisan Formation from the bottom of the Upper Gypsum Unit (UGU; ~ 3.8 m) to the top of the Additional Gypsum Unit (AGU; 0 m) that form the terminal deposit at this site. The Lisan Formation at Masada consists of aad varves e.g.^17,52^ and several massive gypsum deposits e.g.^15^. About 1.37 m from the uppermost UGU to the lowermost AGU (Fig. 3a,c–h) were sampled continuously with overlap in 2018. After smoothing the outcrop surface with a sharp knife, stainless steel boxes (~ 34 cm × 5 cm) with removable side walls were pressed along a vertical profile into the sediment with an overlap of several centimeters. A battery-operated dovetail saw was used to cut the hard gypsum sections. The majority of the gypsum units were not sampled for microfacies analyses because the gypsum is too hard and brittle for thin section preparation. However, 3 cm from the top of the UGU and 18 cm from the bottom of the AGU were recovered and provide basic microfacies data even from gypsum deposits. Because the sampling site is located in an arid region, the sediments were sampled dry, and were not dried before further treatment at the GFZ in Potsdam. The sediment was carefully transferred into aluminum boxes and impregnated with epoxy resin. Then, samples were cut into two halves—one half was impregnated with epoxy resin again and the second half was used for thin section preparation (10 × 2 cm with 2 cm overlap).

### Microfacies analyses and varve chronology

At both sites, the study intervals for microscopic investigation were sampled continuously for thin sections following the standard procedure by^53^ that was adjusted for salty sediments. In total 129 thin sections were prepared, 109 from site 5017-1-A, and 20 from Masada. Thin section analyses were performed using a Zeiss Axiolab pol microscope under plane- and cross-polarized light using magnifications of 50-400x. Photographs were taken with an Olympus BX53F microscope, connected to an Olympus DP72 camera with magnifications between 20- and 400 times. Microfacies analyses included varve counting and measurements of varves and sublayer thickness that were conducted based on determination of varve composition, structure and boundaries. A varve quality index (VQI) from 0 (no varve preservation) to 3 (perfect horizontal varve with sharp boundaries) was assigned to each varve. At both sites, floating varve chronologies were established for lithozones C2 and M2 by microscopic layer counting.

Counting was performed two (C2) and three (M2) times and the sublayer thickness was measured during the second count, which is considered more reliable^54^. The counting difference between the counts was calculated for each thin section. Overcounts (+ varves) and undercounts (-varves) are given as counting uncertainty. In lithozone M2, varves were only counted, but not measured in five folded intervals caused by earthquakes^55^, similar to the method of Prasad et al. (2004)^52^.

### XRF analyses

XRF core scanning was performed on smoothed surfaces of fresh sediment from cores 5017–1-A-46 to -43 with an ITRAX XRF core scanner at the GFZ in Potsdam using a Cr-X-ray source (30 kV, 30 mA), 10 s measurement time and a measurement step size of 200 µm. Element intensities are acquired in counts per second (cps) and displayed as log-ratios reflecting relative variations of the geochemical composition in the ICDP sediment cores^56–58^.

µ-XRF element mapping was performed on the sedimentary sections collected from both sites—the ICDP core and Masada—on selected impregnated sediment blocks that were also utilized for thin section preparation. The µ-XRF element mapping was performed at the GFZ in Potsdam using a Bruker M4 Tornado µ-XRF scanner. The scanner is equipped with a Rh X-ray source (50 kV, 600 µA) and poly-capillary X-ray optics, which irradiate a spot size of approximately 20 µm. Using a measurement time of 30 ms, measurements were obtained every 50 µm and relative element abundances are visualized as 2D maps using normalized element intensities. µ-XRF element maps reveal compositional differences at sub-annual resolution, thus directly complementing thin section microscopy.

### Chronology of the ICDP core

We developed a revised chronology based on Bayesian age modelling in OxCal^26–28^ for the lateglacial section of the ICDP core. Our age model includes three tephrochronological ages from Neugebauer et al. (2021)^22^ and three radiocarbon dates from Kitagawa et al. (2017)^23^ (Fig. 2, Table S1). The three cryptotephra horizons were identified between ~ 93.3 and 92.0 m and correlated to (i) the Nemrut V-16 eruption at 13,585 ± 1.4% vy BP^29,30^ (93.26 m), (ii) the Süphan swarm eruptions V-8 to V-15 at 12,740–13,078 ± 1.4% vy BP^29,30^ (92.54 m), (iii) the Santorini PhT1 at ~ 13,900–10,500 BP^59^ (92.07 m) and (iv) the Süphan V-13 eruption at 12,740–13,078 ± 1.4% vy BP^29,30^ (92.07 m). Ages for the Nemrut eruption and Süphan swarm eruptions are derived from varve counting in the Lake Van sediment record^29,30^ and the Santorini PhT1 tephra age is from a radiocarbon dated peat^59^. The relative timing between the two tephra horizons at 93.26 m and 92.54 m is confirmed by independent varve counting. For our age model we accept the Nemrut V-16 tephra, and the Süphan Swarm tephra ages (No. 6, 5 and 3 in Fig. 2b), but exclude the Santorini PhT1 tephra, because it has a too large uncertainty compared with the Süphan V-13 tephra in the same sample.

In addition to three tephra ages, we include three radiocarbon ages^23^ in the age model after re-calibration with IntCal20 in OxCal^60^. (1) The youngest (16,591 ± 131 cal. BP; No. 10 in Fig. 2b) from three ^14^C-ages all derived from the same thick MTD directly below the base of the UGU at 103.07 m. All three radiocarbon ages reveal similar ages around 17 ka (No. 10–8 in Fig. 2b). We have selected the youngest of these ages because possible reworking effects are lowest. (2) The ^14^C-age within the UGU at 98.51 m depth (16,203 ± 100 cal. BP; No. 7 in Fig. 2b). (3) The radiocarbon age from 89.25 m sediment depth ~ 20 cm above the top of the AGU (11,448 ± 122 cal. BP; No. 1 in Fig. 2b). Another radiocarbon age^23^ (14,161 ± 160 cal. BP; No. 2 in Fig. 2b) between the UGU and AGU is obtained from the base of a MTD and is considered as reworked and is ~ 1 ka older than the tephra horizon from the same depth. In summary, our Bayesian age depth model (OxCal v.4.4; P_Sequence (1,1,C(− 2,2))^26–28^ for the ICDP core includes the above described three radiocarbon and three tephra ages (Fig. 2b).

The resulting age model covers a time span of about 5300 years and dates the UGU from 16,449 + 143/− 149 BP to 14,186 + 394/− 459 BP and the AGU from 12,753 + 308/− 276 BP to 11,540 + 151/− 218 BP (age uncertainties are given within the 68.3% confidence interval). The relative duration of ca. 1000 years between the UGU and the AGU is confirmed by independent varve counting both in the ICDP core (912 varves) and Masada (968 varves). The lower part of the age model below ca. 13,500 BP (No. 6, Fig. 2b) is not well-constrained due to only few scattered varves and only two age points. Due to many erosive MTDs and core gaps some information is also missing.

## Supplementary Information

Supplementary Information.

## Data Availability

Data of this article will be publicly available on PANGAEA (https://www.pangaea.de/) and on the VARDA (https://varve.gfz-potsdam.de/) varve data base.
