# Supplementary material for: Phases of stability during major hydroclimate change ending the Last Glacial in the Levant
Source: Sci Rep. 2022 Apr 27;12:6052. doi: 10.1038/s41598-022-10217-9 (PMC9046258; doi:10.1038/s41598-022-10217-9)
Supplement: Supplementary file 1 — Supplementary Information. [file 41598_2022_10217_MOESM1_ESM.docx]

**Supplementary Information**

# **Supplementary Figures**


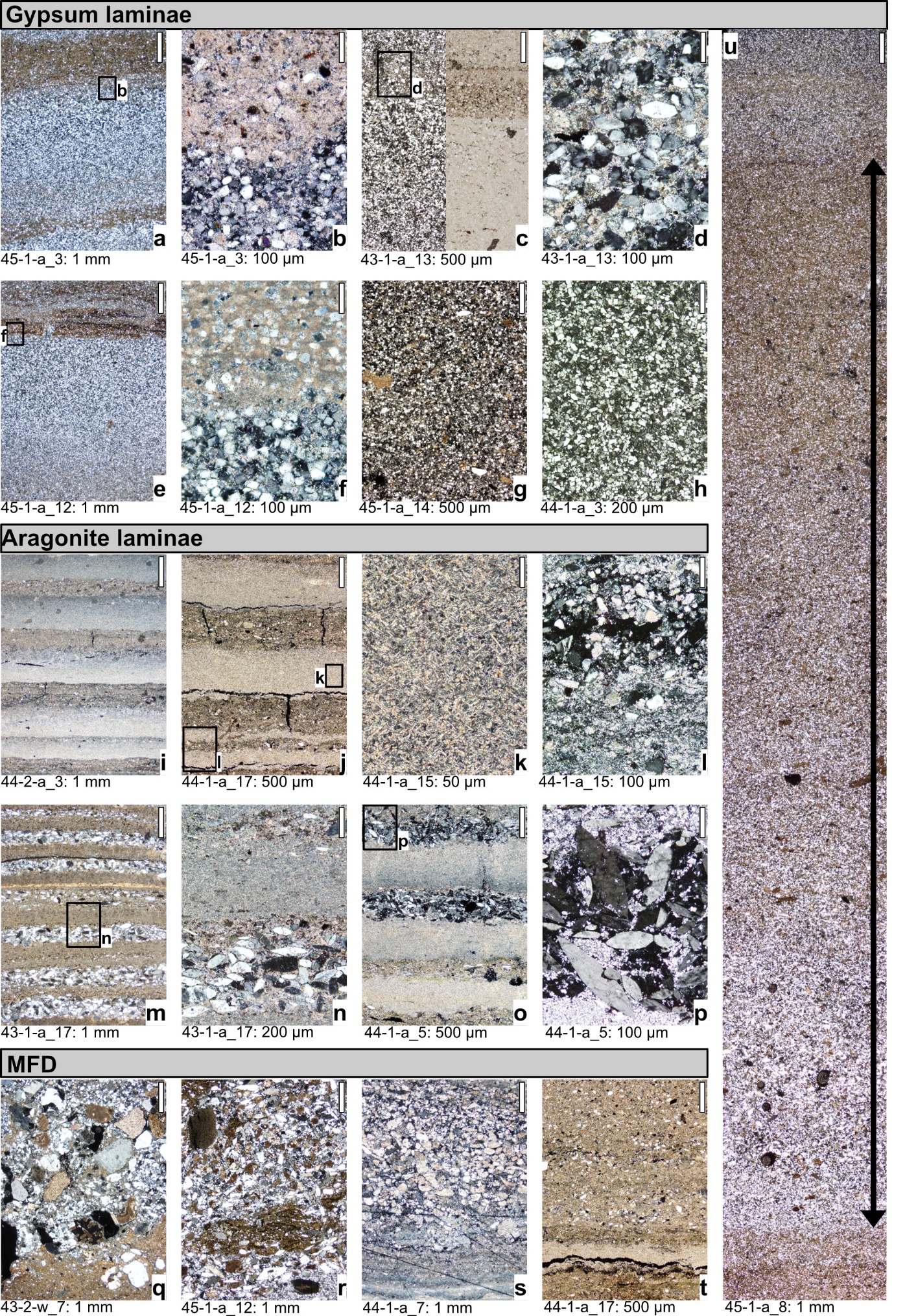


**Figure S1**: Microscope images of the different ICDP facies in our study interval.

(a-h, u) Gypsum microfacies. (a-d) gd-l1 consisting of rectangular gypsum and a thinner fine detrital sublayer; (e, f) gd-l1 consisting of rectangular gypsum and a thinner top of carbonate cement between gypsum grains; (g) gypsum grains overlain by coarser detrital grains; (h) pure fine-grained gypsum ; (u) gd-l1a consisting of rectangular gypsum grains and graded detritus. (i-l) aad varves consisting of one aragonite and one detrital sublayer. (m-p) aadg varves consisting of couplets of aragonite and idiomorphic elongated gypsum grains. (q-t) base of MFDs. (q-s) MFD-1 with sand-sized detrital grains and basal erosion; (t) matrix-supported MFD-2 with clay to silt-sized detrital grains and rare basal erosion. Core names (e.g. ‘45-1-a’) and thin section numbers (e.g. ‘_3’) are given below each image. Note the different scales (white boxes): box scale is specified at the bottom of each picture.


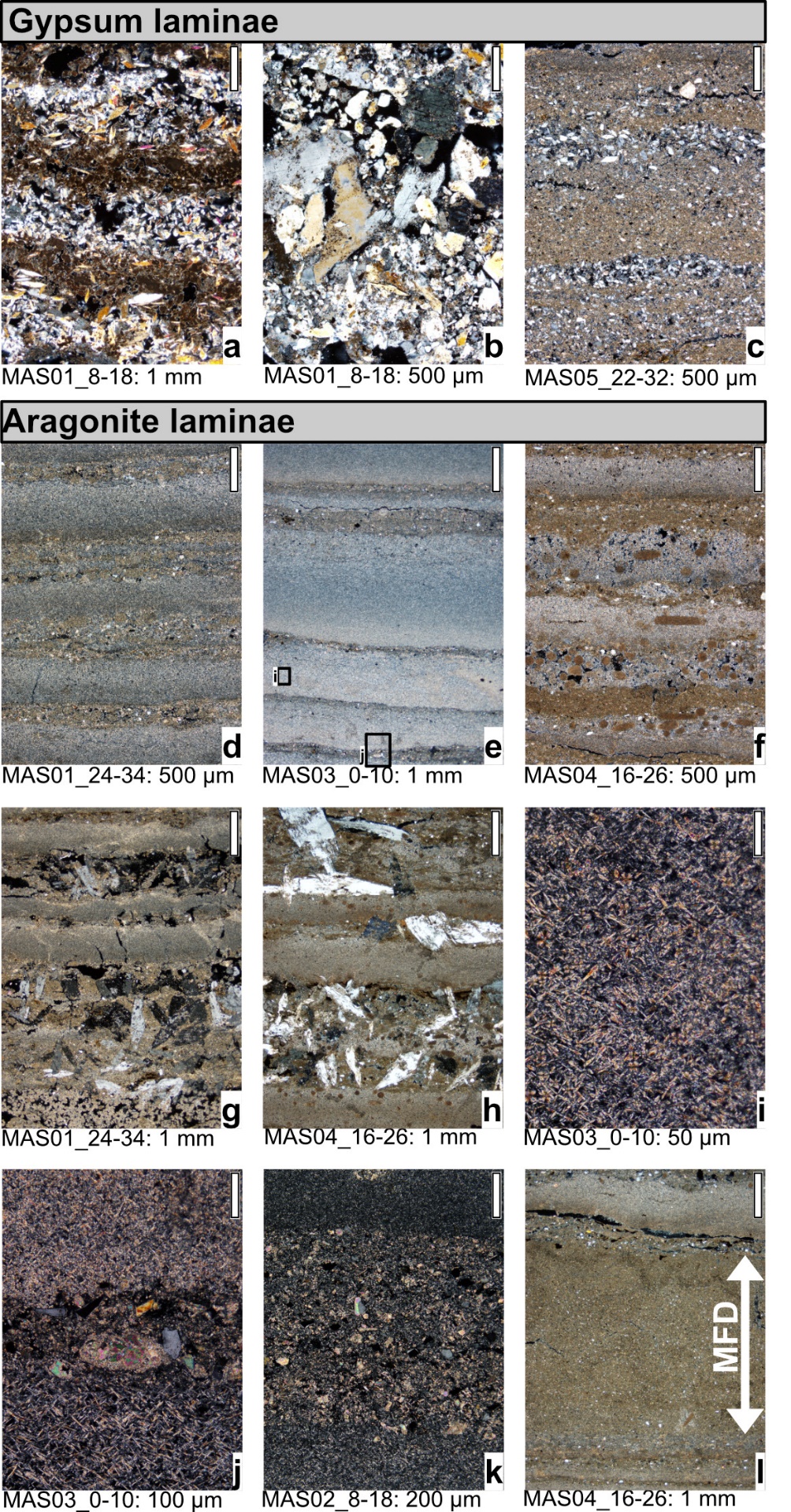


**Figure S2**: Microscope pictures of the Masada facies in our study interval.

(a-c) Gypsum facies gd-l3 (a, b) and gd-l2 (c) consisting of gypsum couplets of light idiomorphic elongated gypsum grains and dark detritus. Note the detrital lumps in (a) and the large diagenetic gypsum grains in (b). (d-k) Variety of aragonite varves. (d-f) aad varves of varying thickness, occasionally including micritic grains (f). (g, h) aadg varves with post-depositional gypsum growth structures. (i-k) Zoom into aragonite varve sublayers: light aragonite (i, j) and dark detritus (j, k). (l) Matrix-supported MFD-2 within aad varves with clay to silt-sized detrital grains lacking erosion. Block names (e.g. ‘MAS01’) and thin section numbers (e.g. ‘_8-18’) are given below each image. Note the different scales (white boxes): box scale is specified at the bottom of each picture.

# **Supplementary Tables**

**Table S1**: Published ages for the ICDP core 5017-1-A and Masada.

^14^C-age calibration with IntCal20^1^. MTD: mass transport deposit, unc.: uncertainty.

| **5017-1-A** | **Age ± unc. (BP)** | **Type of age** | **Material** | **Sediment type** | **Original reference** |
| --- | --- | --- | --- | --- | --- |
| 1 | 11,448 ± 122 | Calibrated ^14^C-age | terrestrial plant remains | MTD | Kitagawa et al. (2017)^2^ |
| 2 | 14,161 ± 160 | Calibrated ^14^C-age | terrestrial plant remains | MTD | Kitagawa et al. (2017)^2^ |
| 3 | ~13,900-10,500 | Tephra (Santorini PhT1) | glass shards | varves + MTD | St. Seymour et al. (2004)^3^; Neugebauer et al. (2021)^4^ |
| 3 | 12,740 - 13,078 ± 1.4% | Tephra (Süphan V-13 eruption) | glass shards | varves + MTD | Landmann et al. (1996)^5^; Schmincke & Sumita (2014)^6^; Neugebauer et al. (2021)^4^ |
| 4 | 14,067 +1146/-1135 | Isochron-U/Th-Age (± 2σ) | aragonite | varves | Torfstein et al. (2015)^7^ |
| 4 | 12,858 ± 363 | Average single sample-U/Th-Age (± 2σ) | aragonite | varves | Torfstein et al. (2015)^7^ |
| 5 | 12,740 - 13,078 ± 1.4% | Tephra (Süphan V-8 to V-15 swarm eruptions) | glass shards | varves + MTD | Landmann et al. (1996)^5^; Schmincke & Sumita (2014)^6^; Neugebauer et al. (2021)^4^ |
| 6 | 13,585 ± 1.4% | Tephra (Nemrut V-16 eruption) | glass shards | varves + MTD | Landmann et al. (1996)^5^; Schmincke & Sumita (2014)^6^; Neugebauer et al. (2021)^4^ |
| 7 | 16,203 ± 100 | Calibrated ^14^C-age | terrestrial plant remains | MTD | Kitagawa et al. (2017)^2^ |
| 8 | 16,854 ± 95 | Calibrated ^14^C-age | terrestrial plant remains | MTD | Kitagawa et al. (2017)^2^ |
| 9 | 17,196 ± 91 | Calibrated ^14^C-age | terrestrial plant remains | MTD | Kitagawa et al. (2017)^2^ |
| 10 | 16,591 ± 131 | Calibrated ^14^C-age | terrestrial plant remains | MTD | Kitagawa et al. (2017)^2^ |
| **Masada** | **Age ± unc. (BP)** | **Type of age** | **Material** | **Sediment** | **Original source** |
| 11 | 14,500 ± 500 | tuned U/Th-Age (± 1σ) | aragonite | varves | Torfstein et al. (2013)^8^ |
| 11 | 13,600 ± 2,200 | Regression-U/Th-Age (± 2σ) | aragonite + organic macrofossils | varves | Torfstein et al. (2013)^8^ + Prasad et al. (2004)^9^ |
| 12 | 15,500 ± 500 | tuned U/Th-Age (± 1σ) | aragonite | varves | Torfstein et al. (2013)^8^ |
| 12 | 15,170 ± 2,200 | Regression-U/Th-Age (± 2σ) | aragonite + organic macrofossils | varves | Torfstein et al. (2013)^8^ + Prasad et al. (2004)^9^ |
| 13 | 17,100 ± 500 | tuned U/Th-Age (± 1σ) | aragonite | varves | Torfstein et al. (2013)^8^ |
| 13 | 17,730 ± 2,200 | Regression-U/Th-Age (± 2σ) | aragonite + organic macrofossils | varves | Torfstein et al. (2013)^8^ + Prasad et al. (2004)^9^ |

**Table S2**: Microfacies data for the lithozones C1-C4 (ICDP core) and M1-M3 (Masada).

| **ICDP core 5017-1-A** | | | | | |
| --- | --- | --- | --- | --- | --- |
| Lithozones |  | **C1** | **C2** | **C3** | **C4** |
| Unit |  | **UGU** | **ULi-aad** | **AGU** |  |
| Depth (m) |  | 100.94-94.69 | 94.69-91.76 | 91.76-89.46 | 89.46-88.48 |
| Thickness (m) |  | 6.25 | 2.93 | 2.29 | 0.98 |
| Gypsum | Sum of beds | 1.40 m (32%) | 0.09 (3%) | 0.36 m (21 %) | - |
|  | No. Of beds (#) | 7 | 2 | 4 | - |
|  | Individual beds | Up to 33 cm, Up to ~100 laminae | Up to ~10 cm, up to ~10 laminae | ~10-20 cm (laminated lower beds), ~4 cm (upper beds, ~42 % laminated, ~58 % massive) | - |
|  | Gypsum laminae # (thickness %) | 224 (97%) | 16 (100%) | 53 (42%) | - |
|  | Gypsum massive (thickness %) | 3% | - | 58% | - |
|  | Gypsum laminae type | gd-l1 | gd-l1 | gd-l1 | - |
| Aragonite varves | Sum of aad/aadg packages | 0.21 m (5%) | 0.76 m (27%) | 0.23 m (13 %) | 0.08 m (8%) |
|  | aragonite varves No. (#) | 339 | 912 | 208 | 50 |
|  | aad varves (#) | 199 | 848 | 102 | 2 |
|  | aadg varves (#) | 50 | 64 | 106 | 48 |
|  | Sum of ld packages | 0.19 m (4 %) | - | - | - |
|  | ld laminae (#) | 90 | - | - | - |
|  | Max. varves without MTD (#) | 36 | 54 | 39 | 23 |
|  | aragonite varves between gypsum beds | 25-231 | - | 208 | - |
| MTD | Sum of MTDs | 2.60 m (59 %) | 1.94 m (70 %) | 1.15 m (66 %) | 0.91 (92%) |
|  | MTD No. (#) | 47 | 64 | 27 | 11 |
|  | MTD erosive No. (#) | 20 (43%) | 13 (20%) | 6 (22%) | 4 (36%) |
|  | Sum of MFDs | 2.26 m (51%) | 1.51 m (54%) | 1.10 m (63%) | 0.91 (92%) |
|  | MFD No. (#) | 43 | 61 | 26 | 11 |
|  | MFD-1 No. (#) | 16 (37%) | 6 (10 %) | 4 (15%) | 4 (36%) |
|  | MFD-1 max. thickness (m) | 0.58 m | 0.15 m | 0.49 m | 0.64 m |
|  | MFD-2 No. (#) | 27 (63%) | 55 (90%) | 22 (85%) | 7 (64%) |
|  | MFD-2 max. thickness (m) | 0.04 m | 0.08 m | 0.04 m | 0.02 m |
|  |  |  |  |  |  |
|  |  |  |  |  |  |
|  |  |  |  |  |  |
| **Table S2 (continued)**: Microfacies data for the lithozones C1-C4 (ICDP core) and M1-M3 (Masada). | | | | | |
| **Masada** | | | | | |
| Lithozones |  | **M1** | **M2** | **M3** | **-** |
| Units |  | **UGU** | **ULi-aad** | **AGU** | **-** |
| Depth (m) |  | 3.8-1.55 | 1.55-0.41 | 0.41-0 | - |
| Thickness (m) |  | 2.29 | 1.16 | 0.41 | - |
| Microscopic analyses |  | 1.6-1.55 | 1.55-0.41 | 0.41-0.23 | - |
| Gypsum | Sum of beds | ~0.85 m* (37%) | - | 0.41 m (100%) | - |
|  | No. Of beds (#) | ~9* | - | 1 | - |
|  | Individual beds | - | - | - | - |
|  | Gypsum laminae (#) | - | - | - | - |
|  | Gypsum type | gd-l2 | - | gd-l3 | - |
| Aragonite varves | Sum of aad packages | - | 1.13 (97%) | - | - |
|  | aragonite varves No. (#) | - | 968 | - | - |
|  | aad varves (#) | - | 926 | - | - |
|  | aadg varves (#) | - | 42 | - | - |
|  | Sum of ld packages | - | - | - | - |
|  | ld laminae (#) | - | - | - | - |
|  | Max. varve No. without MTD (#) | - | 7-297 | - | - |
|  | aragonite varves between gypsum beds | - | - | - | - |
| MTD | Sum of MTDs | - | 0.03 (3%) | - | - |
|  | MTD No. (#) | - | 12 | - | - |
|  | MTD erosive No. (#) | - | 0 (0 %) | - | - |
|  | Sum of MFDs | - | 0.03 (3%) | - | - |
|  | MFD No. (#) | - | 12 | - | - |
|  | MFD-1 No. (#) | - | - | - | - |
|  | MFD-1 max. thickness (m) | - | - | - | - |
|  | MFD-2 No. (#) | - | 12 (100%) | - | - |
|  | MFD-2 max. thickness (m) | - | 0.08 m | - | - |
| - does not occur  * data from ^10^ | | | | | |

**Table S3**: P. values for each varve group for the total varve thickness, aragonite sublayer (SL) thickness and detritus+gypsum SL thickness. Groups G1-G3 (in group_1) represent phases of higher aragonite SL thickness at both sites, whereas groups B (in group_2) represent the summarized intervals of lower aragonite SL thickness for each site. In order to analyze differences between groups across the studied segments, each pair of varve groups was compared using the Mann-Whitney-Wilcoxon rank-sum test (Table S3)^11^. The presented p. values are corrected for the false discovery rate of multiple comparisons, applied for every series of tests comparing groups by each of the analyzed parameters (e.g. varve thickness, aragonite SL thickness etc.)^12^. Differences between groups with resulting p. values smaller than 0.05 are considered statistically significant considering a confidence level of 0.95.

| Site | Group_1 | Group_2 | VarveThickness_mm_ | AragoniteSL Thickness_mm_ | Detritus_GypsumSL  Thickness_mm_ |
| --- | --- | --- | --- | --- | --- |
| Masada | G1_Masada | B_Masada | 0.001232091 | 4.62657E-06 | 0.936531406 |
| Masada | G2_Masada | B_Masada | 0.402291506 | 0.117854245 | 0.533865375 |
| Masada | G3_Masada | B_Masada | 7.96865E-05 | 2.03763E-08 | 0.936531406 |
| Masada | G1+G2+G3_Masada | B_Masada | 3.16888E-06 | 7.08057E-11 | 0.936531406 |
| Masada | G1+G3_Masada | B_Masada | 3.16888E-06 | 7.08057E-11 | 0.969672604 |
| ICDP | G1_ICDP | B_ICDP | 0.006771545 | 2.24138E-08 | 0.084622943 |
| ICDP | G2_ICDP | B_ICDP | 0.802073451 | 0.54624158 | 0.817974023 |
| ICDP | G3_ICDP | B_ICDP | 0.011363016 | 1.16867E-06 | 0.031644052 |
| ICDP | G1+G2+G3_ICDP | B_ICDP | 0.001539175 | 3.48498E-10 | 0.01218223 |
| ICDP | G1+G3_ICDP | B_ICDP | 0.001232091 | 1.07549E-10 | 0.01218223 |

# **Sedimentary facies**

Sediments in our study sections of the ICDP core and Masada consist of three different types of aragonite varves^e.g. 9,13^, four different types of mass transport deposits (MTD)^14^ and five different types of gypsum facies.

## Gypsum

Gypsum appears in different facies including discrete gypsum deposits, MTDs and in aragonite laminae at both sites. Interpretation of gypsum deposition is based on the grain forms, grain orientation and microstratigraphic position. We differentiate two crystal forms – idiomorphic elongated and interlocking rectangular/prismatic. Gypsum is found predominantly in various laminated facies and only rarely non-laminated and massive. We distinguish laminated gypsum facies from the deep-water site (lithozones C1, C2 and C3) and the shallow water site (lithozones M1 and M3).

Laminated gypsum facies 1 (gd-l1; Fig. S1a-h, u) dominates in lithozones C1, C2 and C3 of the ICDP core (73 %, 215 couplets) and consists of two alternating sublayers (i) yellow, interlocking rectangular gypsum grains and rarely elongated horizontally orientated grains and (ii) (dark-) brownish fine-grained detritus (Fig. S1a-d). The grain forms in the gypsum sublayer are indicative for gypsum precipitation in the water column, which is supported by inverse grading of some of these gypsum layers^15–18^. The detrital sublayer consists of a clay-sized marl matrix, which includes scattered rectangular, likely reworked, gypsum grains. Detrital sublayers occasionally exhibit carbonaceous cementation (Fig. S1e, f) suggesting early diagenetic re-crystallization. Similar gypsum laminations in Australian lake sediments were tentatively related to an annual cycle (varves)^15^. However, due to the lack of modern observation of such depositional processes this can neither be confirmed nor disproven for the Dead Sea gypsum laminations. In lithozones C1 and C2, these laminations are occasionally intercalated by ~1-3 cm thick, partly graded mixed detrital/gypsum layers (in total, 15 layers, sub-type gd-l1a, Fig. S1u). The basal, coarser grained part of these layers is enriched in gypsum while fine-grained marl becomes dominant towards the finer-grained top. These layers are interpreted as runoff-triggered event deposits, which may include even some rectangular precipitated gypsum grains^15–17^.

Laminated gypsum facies 2 (gd-l2; Fig. S2c) occurs in lithozone M1 (UGU, Masada) and resembles gd-l1 in the general sublayer structure, but differs with respect to gypsum grain shapes. Gypsum sublayers consist of mostly elongated and horizontally orientated gypsum grains suggesting re-deposition. The detrital sublayer consists of a fine-grained marl matrix and coarser detrital grains than gd-l1, including scattered elongated gypsum grains.

Laminated gypsum facies 3 (gd-l3; Fig. S2a, b) occurs in lithozone M3 (AGU, Masada). The couplet structure resembles that of gd-l2, but clearly differs by the frequent occurrence of large (up to 2 mm) gypsum crystals indicative for post-depositional growth^15,16^ in both sublayers and often interlocking rectangular precipitated gypsum grains^15–17^ in the basal gypsum sublayer. Another difference to gd-l1 and-l2 is the occurrence of fine-grained lumps in the detrital layers, which suggests shallow-water or aeolian^15,16^ deposition.

The observed differences in laminated gypsum facies between the Masada outcrop and the ICDP core nicely reflects the different sedimentary environments in shallow- and deep-water environments. In the shallow-water site Masada, the dominance of reworked and diagenetic gypsum alternating with sublayers consisting of fine-grained lumps of detrital matter, as well as undulating layer boundaries indicate a shallow-water environment influenced by wave activity^19,20^ or even seasonal exposure and aeolian reworking/deposition^15,16^. In contrast, the deep-water sedimentation is characterized by gypsum formed in the water column and, less common, by re-deposition through event-triggered sedimentation.

Massive gypsum deposits show no internal structure and occur in ICDP core lithozones C1 (~4.6 cm) and C3 (~11 cm). These deposits largely consist of yellow, interlocked rectangular and elongated gypsum, but also include detrital carbonates, carbonate fossils, micritic grains, aragonite needles and clay-sized marl. Gypsum occurs as precipitated idiomorphic crystals^15–18^.

Additionally, gypsum occurs in MTDs and in aragonite laminae at both sites. In the ICDP core ‘aadg’ varves, elongated gypsum grains occur in horizontal sublayers (Fig. S1m-p) suggesting either reworking^15,20^ or slow precipitation from the water column^16^. In contrast, in ‘aadg’ varves at Masada large idiomorphic gypsum crystals and displacement structures (Fig. S2g, h) indicate post-depositional formation.

Reworked gypsum^e.g. 15^ is found in MFD’s in the ICDP core, where it predominantly occurs in the coarser basal parts and in lower portions also in the upward fining part of the MFD’s (Fig. S1q-t). In the Masada profile, rarely early-diagenetic gypsum^15,16^ appears especially in the coarse grained basal part of the MFD’s.

## Aragonite varves

Aragonite varves occur at both sites and are differentiated into three subtypes in the ICDP core and two subtypes in Masada. Alternating aragonite and detritus (aad) varves^e.g. 9,13,14,21^ occur in all ICDP core lithozones (Fig. S1i-l) and in lithozone M2 in Masada (Fig. S2d-f). In the ICDP core ~76 % (1151 couplets) and in Masada ~96% (926 couplets) of all aragonite laminae are typical aad varves. The thickness of these couplets is up to 6.8 mm in the ICDP core and up to 4.8 mm in Masada. The aad facies typically consists of two laminae: (i) white authigenic aragonite needles or stellate aggregates of orthorhombic crystals (~5-30 µm; Figs S1k, S2i, j) and (ii) dark allochthonous clay to silt-sized detrital carbonates, quartz, feldspar and clay minerals (Figs S1i, j, l; S2j, k)^e.g. 22,23^. Primary aragonite precipitates during the dry season due to evaporation, while the detritus is deposited by seasonal floods during the rainy season^22,24^.

A subtype of aad varves (termed ‘aadg’ varves here) occurs in all lithozones of the ICDP core (Fig. S1m-p) and in lithozone M2 at Masada (Fig. S2g, h). The aadg facies occurs in 18 % (268 laminae) of aragonite varves in the ICDP core and in ~4 % (42 laminae) of aragonite varves in Masada. The thickness of these couplets is up to 7 mm in the ICDP core and up to 3.5 mm in Masada. Sublayers are identical to aad varves except for the incorporation of idiomorphic elongated gypsum grains (section S1.1) following the aragonite sublayer or in the detrital sublayer. At Masada gypsum grains also occur within the aragonite sublayer in 21 aadg varves (in previous works referred to as ‘disseminated gypsum’^10^).

A rare aragonite varve subtype termed ld (‘laminated detritus’^14,23^) occurs solely in lithozone C1 of the ICDP core and amounts to ~6 % (90 laminae) of aragonite varves. The thickness of these laminae is up to 2.7 mm. Two sublayers comprise (i) finer detritus and (ii) coarser grained detritus often incorporating diagenetic elongated gypsum^15^. Rarely, a third sublayer consisting of aragonite is developed.

## Mass Transport Deposits

We differentiate MTDs based on previous descriptions^14,25–28^ into (i) mass flow deposits (MFD), (ii) homogenites, (iii) slumps and (iv) breccias at both sites. In the ICDP core, between 20 and 43 % of MTDs show erosive structures at their base, whereas at Masada no erosive structure are observed (Table S2). Mass flow deposits (MFD) are the most common MTDs (Table S2) and describe detrital sediments with a coarse sand to silt-sized base and upwards fining. We differentiated two types. MFD-1 (Fig. S1q-s) has an erosional base and typically consist of three sections with decreasing detrital grain sizes and quantity (thickness up to ~64 cm). The base is grain-supported, poorly sorted and encompasses up to sand-sized angular to rounded detrital grains (up to 2 mm; carbonates, reworked gypsum and sub-ordinated quartz, feldspar and organic remains). This MFD type occurs solely in the ICDP core. MFD-2 (Figs S1t, S2l) consists of one matrix-supported layer (thickness up to ~8 cm in the ICDP core and up to ~0.8 cm at Masada) with smaller (up to ~0.1 mm) detrital grains and rarely show basal erosion. They either have a coarse base, are fining upwards or are homogenous. MFD-2 are the only MTD occurring at Masada. MFDs in the ICDP core mostly incorporate elongated idiomorphic gypsum interpreted as reworked (section S1.1)^15^. The ICDP core comprises 141 MFDs (30 MFD-1 and 111 MFD-2) with 38 (including all MFD-1) showing erosive structures (e.g. undulating base, dropped grains, half-eroded underlying sublayers). At Masada occur 12 MFD-2 (~0.8 to ~8.4 mm thickness) that show no visible basal erosion.

Homogenites occur only in lithozones C1 and C2 in the ICDP core and are defined as matrix-supported deposits of coarse scattered detrital grains in a marl matrix. They occur three times and are 5-36 cm thick. Due to a missing bottom boundary one could also be defined as MFD. Slumps occur solely in lithozones C2 and C3 of the ICDP core and are in-situ aad laminae that are large-scale folded, fractured and/or dislocated within homogeneous sediments interpreted as subaquatic slope failures. These occur two times and are ~5 cm thick. Breccias, i.e. deposits of broken aad laminae fragments within a fine-grained dark matrix, appear only in lithozones C1 and C2 of the ICDP core. Either they occur as discrete deposit or are incorporated in the base of MFD-1. Discrete breccia deposits appear three times and are 2-2.5 cm thick.

# **Aragonite varve counting and chronologies**

In lithozone C1 of the ICDP core, 339 +10/-18 aragonite varves, including aad, aadg and ld laminae, were counted. A floating varve chronology that consists of in total 912 +15/-24 aad/aadg varves (Table S2) could be constructed solely for lithozone C2 (uncertainties in Fig. 2a are according to uncertainties of the anchor age and in the varve counting). The chronology further shows two intervals of statistically significantly higher aragonite sublayer thickness that are ~110 and ~200 years long (Fig. 4d; Table S3). The varve thickness ranges from 0.03 to 6.8 mm (mean 0.82 mm) and the aragonite sublayer thickness varies between 0.04 and 1.85 mm (mean 0.63 mm). Here, the varve chronology is interrupted 11 times by erosional events and therefore, an unknown number of eroded varves must be assumed. For lithozone C3, 208 +9/-13 aad/aadg varves and for lithozone C4, 50 +10/-0 aad/aadg varves were counted. A total of 968 +15/-64 aad and aadg varves were counted in lithozone M2 at Masada (Table S2). Similar to lithozone C2 in the ICDP core, two ~140-year long intervals of statistically significant higher aragonite sublayer thickness occur (Fig. 4b; Table S3). The varve thickness varies between 0.11 and 4.81 mm (mean 1.1 mm) and the thickness of the aragonite sublayer ranges from between 0.04 to 3.2 mm (mean 0.39 mm). In general, varve thicknesses in the deep-water ICDP core have a higher range/amplitude, but thicker varves form more often in the shallow-water at Masada.

# **Gypsum layer counting**

The studied interval of the ICDP core comprises 224 gypsum laminae in C1 (mean thickness ~2.8 mm), 16 gypsum laminae in C2 (mean thickness ~5.4 mm) and 53 gypsum laminae in C3 (mean thickness ~2.9 mm). Due to disturbances, low preservation and indistinct sublayers in the lower part of C3, counting in this section should be considered with care. For details see Table S2.

# **XRF analyses**

Several element ratios representing the key sedimentary features of our study interval were selected. The log(Ti/Ca) ratio represents input of terrestrial siliciclastic material. Calcium occurring in carbonates and gypsum can be either of detrital or authigenic sources, whereas Ti is exclusively detrital. log(Sr/Ca) reflects aragonite formation since Ca occurs in all carbonates including aragonite and calcite, as well as in gypsum, whereas Sr is only abundant in aragonite. The log(S/Ca) ratio represents gypsum, because S content is elevated in gypsum, but is absent in Ca-carbonate minerals. Gypsum deposits are therefore characterized by high log(S/Ca) ratios, and low log(Ti/Ca) and log(Sr/Ca) ratios. log(S/Ca) ratios are highest in gypsum-dominated segments, second highest in the base of MFDs containing reworked idiomorphic elongated gypsum grains, and third highest in aadg varves comprising diagenetic idiomorphic elongated gypsum (Fig. 3q). Gypsum deposition is indicated in element maps by Ca and S (orange color, Fig. 4e). Aragonite varves show a distinct reversed pattern than observed in gypsum deposits: high log(Ti/Ca) and log(Sr/Ca) ratios, and low log(S/Ca) ratios, with aadg varves being the only exception as all of these ratios are high in this subtype (Fig. 3o-q). In element maps, the alternating aragonite (blue Ca and Sr) and detritus (yellow Si) sublayers from aad varves are clearly distinguishable (Fig. 4e, f), and in aadg varves, the gypsum-bearing sublayer is characterized by orange Ca and S maps (Fig. 4e).

# **Supplementary references**

1. Reimer, P. J. *et al.* The IntCal20 Northern Hemisphere Radiocarbon Age Calibration Curve (0-55 cal kBP). *Radiocarbon* **62**, 725–757 (2020).

2. Kitagawa, H., Stein, M., Goldstein, S. L., Nakamura, T. & Lazar, B. Radiocarbon Chronology of the DSDDP Core at the Deepest Floor of the Dead Sea. *Radiocarbon* **59**, 383–394 (2017).

3. St. Seymour, K. *et al.* Tephrostratigraphy and tephrochronology in the Philippi peat basin, Macedonia, Northern Hellas (Greece). *Quat. Int.* **121**, 53–65 (2004).

4. Neugebauer, I. *et al.* Cryptotephras in the Lateglacial ICDP Dead Sea sediment record and their implications for chronology. *Boreas* **50**, 844–861 (2021).

5. Landmann, G., Reimer, A., Lemcke, G. & Kempe, S. Dating Late Glacial abrupt climate changes in the 14,570 yr long continuous varve record of Lake Van, Turkey. *Palaeogeogr. Palaeoclimatol. Palaeoecol.* **122**, 107–118 (1996).

6. Schmincke, H.-U. & Sumita, M. Impact of volcanism on the evolution of Lake Van (eastern Anatolia) III: Periodic (Nemrut) vs. episodic (Süphan) explosive eruptions and climate forcing reflected in a tephra gap between ca. 14 ka and ca. 30 ka. *J. Volcanol. Geotherm. Res.* **285**, 195–213 (2014).

7. Torfstein, A. *et al.* Dead Sea drawdown and monsoonal impacts in the Levant during the last interglacial. *Earth Planet. Sci. Lett.* **412**, 235–244 (2015).

8. Torfstein, A., Goldstein, S. L., Kagan, E. J. & Stein, M. Integrated multi-site U-Th chronology of the last glacial Lake Lisan. *Geochim. Cosmochim. Acta* **104**, 210–231 (2013).

9. Prasad, S. *et al.* Evidence from Lake Lisan of solar influence on decadal- To centennial-scale climate variability during marine oxygen isotope stage 2. *Geology* **32**, 581–584 (2004).

10. Torfstein, A., Gavrieli, I., Katz, A., Kolodny, Y. & Stein, M. Gypsum as a monitor of the paleo-limnological-hydrological conditions in Lake Lisan and the Dead Sea. *Geochim. Cosmochim. Acta* **72**, 2491–2509 (2008).

11. Mann, H. B. & Whitney, D. R. On a Test of Whether one of Two Random Variables is Stochastically Larger than the Other. *Ann. Math. Stat.* **18**, 50–60 (1947).

12. Benjamini, Y. & Hochberg, Y. Controlling the False Discovery Rate: A Practical and Powerful Approach to Multiple Testing. *J. R. Stat. Soc. Ser. B* **57**, 289–300 (1995).

13. Machlus, M., Enzel, Y., Goldstein, S. L., Marco, S. & Stein, M. Reconstructing low levels of Lake Lisan by correlating fan-delta and lacustrine deposits. *Quat. Int.* **73**–**74**, 137–144 (2000).

14. Neugebauer, I. *et al.* Lithology of the long sediment record recovered by the ICDP Dead Sea Deep Drilling Project (DSDDP). *Quat. Sci. Rev.* **102**, 149–165 (2014).

15. Magee, J. W. Late Quaternary lacustrine, groundwater, aeolian and pedogenic gypsum in the Prungle Lakes, southeastern Australia. *Palaeogeogr. Palaeoclimatol. Palaeoecol.* **84**, 3–42 (1991).

16. Mees, F., Castenẽda, C., Herrero, J. & Van Ranst, E. The Nature and Significance of Variations in Gypsum Crystal Morphology in Dry Lake Basins. *J. Sediment. Res.* **82**, 37–52 (2012).

17. Kirkland, D. W. An explanation for the varves of the Castile evaporites (Upper Permian), Texas and New Mexico, USA. *Sedimentology* **50**, 899–920 (2003).

18. Reiss, A. G. *et al.* Gypsum Precipitation under Saline Conditions: Thermodynamics, Kinetics, morphology, and Size Distribution. *Minerals* **11**, 1–38 (2021).

19. Weber, N., Lazar, B., Gavrieli, I., Yechieli, Y. & Stein, M. Gypsum Deltas at the Holocene Dead Sea linked to Grand Solar Minima. *Geophys. Res. Lett.* **48**, 1–10 (2021).

20. Warren, J. K. The hydrological setting, occurrence and significance of gypsum in late Quaternary salt lakes in South Australia. *Sedimentology* **29**, 609–637 (1982).

21. Ben Dor, Y. *et al.* Varves of the Dead Sea sedimentary record. *Quat. Sci. Rev.* **215**, 173–184 (2019).

22. Begin, Z. B., Ehrlich, A. & Nathan, Y. *Lake Lisan: the Pleistocene Precursor of the Dead Sea*. vol. 63 (Ministry of Commerce and Industry, Geological Survey of Israel, 1974).

23. Haliva-Cohen, A., Stein, M., Goldstein, S. L., Sandler, A. & Starinsky, A. Sources and transport routes of fine detritus material to the Late Quaternary Dead Sea basin. *Quat. Sci. Rev.* **50**, 55–70 (2012).

24. Neev, D. & Emery, K. O. *The Dead Sea. Depositional Processes and Environments of Evaporites*. (Monson Press, 1967).

25. Neugebauer, I. *et al.* Hydroclimatic variability in the Levant during the early last glacial ( ∼ 117-75 ka) derived from micro-facies analyses of deep Dead Sea sediments. *Clim. Past* **12**, 75–90 (2016).

26. Neugebauer, I. *et al.* Evidences for centennial dry periods at ~3300 and ~2800 cal. yr BP from micro-facies analyses of the Dead Sea sediments. *The Holocene* **25**, 1358–1371 (2015).

27. Kagan, E., Stein, M. & Marco, S. Integrated Paleoseismic Chronology of the Last Glacial Lake Lisan: From Lake Margin Seismites to Deep-Lake Mass Transport Deposits. *J. Geophys. Res. Solid Earth* **123**, 2806–2824 (2018).

28. Ahlborn, M. *et al.* Increased frequency of torrential rainstorms during a regional late Holocene eastern Mediterranean drought. *Quat. Res. (United States)* **89**, 425–431 (2018).
